# Poly-L-arginine Coated Silver Nanoprisms and Their Anti-Bacterial Properties

**DOI:** 10.3390/nano7100296

**Published:** 2017-09-27

**Authors:** Fouzia Tanvir, Atif Yaqub, Shazia Tanvir, William A. Anderson

**Affiliations:** 1 Department of Zoology, Government College University, Lahore 54000, Pakistan; tanvir.fouzia@gmail.com (F.T.); atif@gcu.edu.pk (A.Y.); 2 Department of Chemical Engineering, University of Waterloo, Waterloo, ON N2L 3G1, Canada; s2tanvir@uwaterloo.ca

**Keywords:** silver nanoparticles, anti-bacterial, poly-L-arginine, stability

## Abstract

The aim of this study was to test the effect of two different morphologies of silver nanoparticles, spheres, and prisms, on their antibacterial properties when coated with poly-L-arginine (poly-Arg) to enhance the interactions with cells. Silver nanoparticle solutions were characterized by UV–visible spectroscopy, transmission electron microscopy, dynamic light scattering, zeta potential, as well as antimicrobial tests. These ultimately showed that a prismatic morphology exhibited stronger antimicrobial effects against *Escherichia coli*, *Pseudomonas aeruginosa* and *Salmonella enterica*. The minimum bactericidal concentration was found to be 0.65 μg/mL in the case of a prismatic AgNP-poly-Arg-PVP (silver nanoparticle-poly-L-arginine-polyvinylpyrrolidone) nanocomposite. The anticancer cell activity of the silver nanoparticles was also studied, where the maximum effect against a HeLa cell line was 80% mortality with a prismatic AgNP-poly-Arg-PVP nanocomposite at a concentration of 11 μg/mL. The antimicrobial activity of these silver nanocomposites demonstrates the potential of such coated silver nanoparticles in the area of nano-medicine.

## 1. Introduction

Silver has been known to have strong inhibitory or bactericidal effects, with a broad spectrum. Silver nanoparticles (AgNPs) are utilized in an increasing number of medical and other products including cosmetics, textiles, electronics, paints, and water disinfectants due to their antibacterial properties [1]. Silver nanoparticles have also been studied for analytical measurements making use of the shift in the wavelength of these plasmonic nanoparticles when they interact with molecules [2,3]. The concern with the growing problem of multidrug-resistant bacteria and their spread has provided a motivation for the development of new and effective bactericidal agents [4]. The bactericidal activity of AgNPs is considered to be mainly due to the size dependent Ag^+^ leaching from the particles. However, it has been reported that stable nanosilver does have a much lower minimal inhibitory concentration than its dissolved ionic counterpart [5], and silver ions in solution are powerful antimicrobials, but they are easily sequestered by chloride, phosphate, proteins, and other cellular components [6]. 

A fundamental problem for the application of AgNPs is to prepare dispersions with sufficient stability to minimize the aggregation process, because the generation of such aggregates leads to a loss of the antibacterial activity [7]. Studies have shown that the antimicrobial activity of AgNPs is strongly size-dependent [8]. Antibacterial properties of the silver nanoparticles are also dependent on their stability in biological media, the type of coating, and surface charge [9,10,11,12,13]. Therefore, unless the silver particles are properly stabilized and dispersed in the media the bactericidal action cannot be properly controlled and evaluated. The role of Poly-Vinyl Pyrrolidone (PVP) as a protective agent that can effectively alter shape, size, stability, and optical properties of AgNPs has been studied and is one promising alternative [14].

To improve their efficacy and stability in biomedical and other applications, the nanoparticles often need to be coated with non-toxic and non-inflammatory capping agents like collagen, peptides, and biopolymers, preferably without significantly changing their size, shape, and antimicrobial properties [3,15,16,17,18,19]. Some findings concerning the action of AgNPs suggest that the bactericidal mode can be influenced by the stability of the particles [5], and as one example AgNPs conjugated with poly-lysine were less effective when compared to collagen-capped AgNPs due to aggregation. 

Furthermore, the electrostatic attraction between positively charged nanoparticles and negatively charged bacterial cells has been shown to be another important aspect with regard to the antimicrobial activity of the AgNPs [20]. Nanoparticle surface charge is a determining factor of cellular uptake [21,22], and those with cations present on their surface more easily bind and are internalized due to electrostatic interactions with the negatively charged cell surface. Although Gram-positive and Gram-negative bacteria have differences in their membrane structure, most of them have a negative surface charge [23]. The negative charges are present on the cell wall due the presence of either teichoic acid in Gram-positive bacteria or the outer membrane lipopolysaccharide in Gram-negative bacteria [24]. Therefore, it is important to develop strategies for a targeted bactericidal action of the silver nanoparticles by exploiting the presence of such charges. However, some negatively charged silver nanoparticles have also been reported as effective anti-bacterial agents [25,26]. In one example, the linking of calix[n]arenes to Ag nanoparticles may reduce the strongly negative charge of the calix-arene tail groups, thereby aiding in membrane penetration of Ag nanoparticles [26]. The efficacy of negatively charged AgNPs may also be explained by the redox potential of Ag atoms on the NPs surface, which is expected to trigger the generation of free radicals leading to reactive oxygen species production [27,28,29]. 

Several arginine rich peptides are reported in the literature as having the ability to disrupt and form pores in lipid bilayers and bacterial cell walls due to positively charged guanidinium groups [30,31,32]. Poly-L-arginine (poly-Arg) is a polycationic biopolymer that has been used as a gene, protein, or drug delivery agent, or as a coating to promote adhesion. Poly-Arg contains positively charged hydrophilic amino groups even in very alkaline media (pKa > 12) due to the guanidinium group [33]. Thus, the electrostatic interactions between bacterial cells and silver nanoparticles coated with poly-Arg could enhance the antibacterial action.

Therefore, poly-Arg seems to be a promising candidate for the coating of silver nanoparticles and hence, by conjugating poly-Arg to silver NPs, the magnitude of AgNPs interactions with microbial cells might be increased. In this work, the main focus was to establish the role of modified particle morphology, charge, and coating on the antibacterial properties. Silver nanoparticles are less susceptible to being intercepted if properly protected with an appropriate coating agent.

To harness the maximum benefits out of silver nanocomposites as an antibacterial agent, the present study was aimed to test two different shapes of silver nanoparticles, spherical and prismatic, stabilized by polyvinylpyrrolidone (PVP) and coated with poly-Arg against *Escherichia coli*, *Pseudomonas aeruginosa* and *Salmonella enterica*.

## 2. Results

### 2.1. Synthesis and Characterization of Silver Nanoparticles

**Figure 1:**
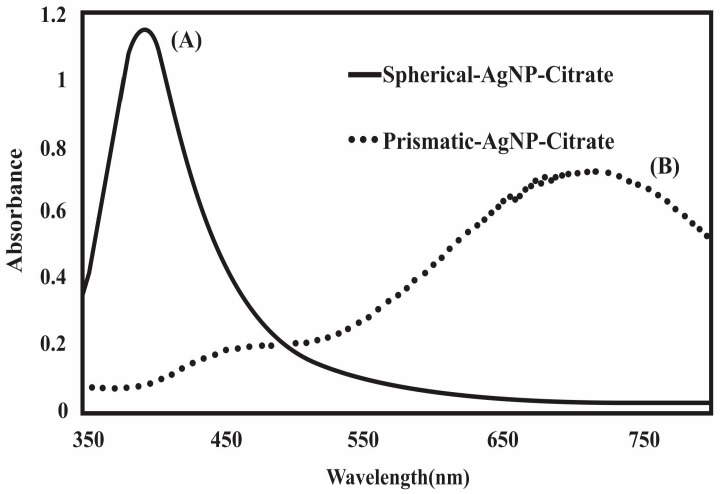
UV-Vis spectra of silver nanoparticles (AgNPs). (**A**) exhibits an intense absorption peak at 400 nm indicating the formation of spherical nanoparticles; (**B**) exhibits an intense absorption peak near 700 nm indicating the formation of prismatic nanoparticles.

Morphologically different types of colloidal silver were prepared to determine the antibacterial activity. Spherical AgNPs were prepared using AgNO_3_, NaBH_4_, and trisodium citrate [34], but using the same reaction mixture nanoprism, AgNPs were synthesized by the addition of H_2_O_2_. This H_2_O_2_ was used to produce oxidative etching in the reaction medium causing significant surface defects in the colloidal silver, such that these clusters would evolve to hexagonal or nanoprism plates [35]. UV-Vis spectra of the yellow (spherical) silver showed an absorption peak at 400 nm (Figure 1A), whereas the blue silver colloid showed an absorption peak at 700 nm, suggesting the formation of nanoprisms (Figure 1B). With the different shapes, colloidal silver possessed very different absorption spectra or color, as a result of multiple resonances in the complex structures [36].

**Figure 2:**
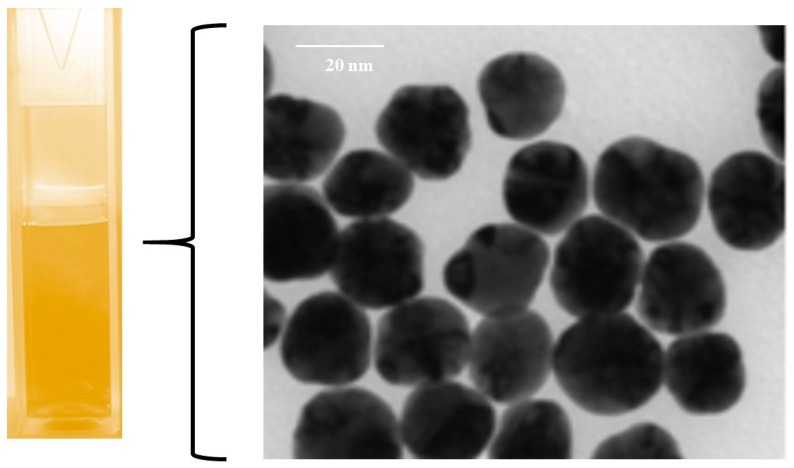
Photograph of uncoated spherical nanoparticles in aqueous suspension (**left**), and Transmission Electron Microscopy (TEM) image of a sample (**right**).

**Figure 3:**
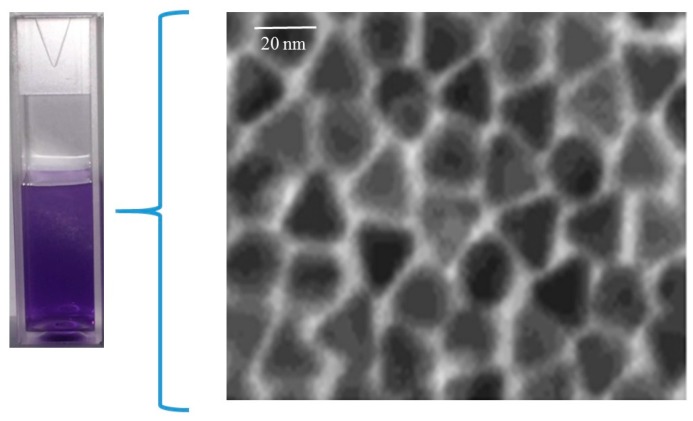
Photograph of uncoated prismatic nanoparticles in aqueous suspension (**left**), and TEM image of a sample (**right**).

**Figure 4:**
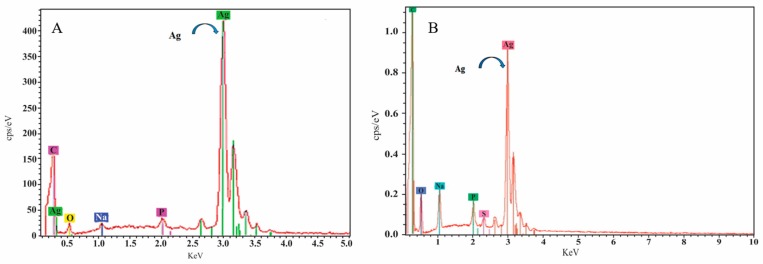
Elemental analysis of spherical nanoparticles (**A**) and prismatic nanoparticles (**B**) performed by energy dispersive X-ray spectroscopy (EDX), confirming elemental silver composition.

The size and morphology of nanoparticles was visualized with Transmission Electron Microscopy (TEM), which confirmed a spherical shape with 20 nm diameter for the yellow silver colloid (Figure 2) while the blue silver showed a nanoprism shape with approximately the same size (Figure 3). The size of the AgNPs was also measured by dynamic light scattering, which showed that the mean diameter of the spherical citrate capped AgNPs was 18.92 nm and that of the prismatic AgNPs was 22.5 nm. The energy-dispersive X-ray spectroscope (EDX) spectra (Figure 4) confirmed that the samples with yellow and blue solutions contained only pure silver.

### 2.2. Coating, Stabilization and Characterization of Silver Nanoparticles

**Figure 5:**
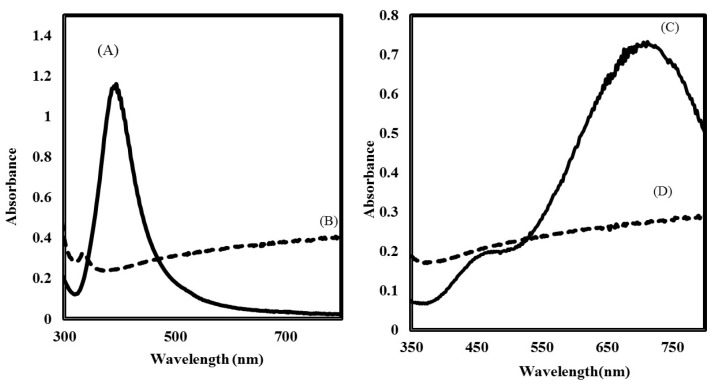
UV-vis absorption spectra of spherical and prismatic AgNPs stabilized with citrate (curve **A** and **C**) and immediately after mixing with 5 mM phosphate buffer saline spiked with nutrient broth (curve **B** and **D**), indicative of the aggregation of colloidal solution under assay conditions.

**Figure 6:**
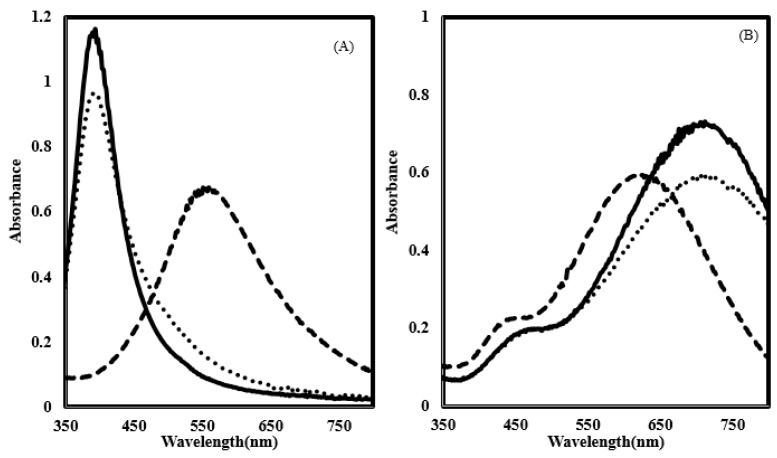
(**A**) Absorption spectra of spherical silver capped with citrate (**─**), polyvinylpyrrolidone (PVP) (**∙∙∙**) and poly-Arg-PVP (**- -**). (**B**) Absorption spectra of prismatic silver capped with citrate (**─**), PVP (**∙∙∙**) and poly-Arg-PVP (**- -**).

The use of surfactants is suggested in the literature for the prevention of aggregation in the biologically relevant medium used to study antibacterial activity [37]. For this purpose, PVP protected colloidal silver was used in this work, since citrate-capped AgNPs rapidly aggregated in the medium during preliminary experiments (Figure 5). In contrast, the UV visible spectra of the PVP protected colloidal silver placed in diluted nutrient broth did not result in peak shifts, suggesting that no significant aggregation occurred. Another strategy applied in this study was to enhance the interaction of AgNPs with the bacterial cells by the functionalization of NPs with poly-Arg first, and then stabilizing with PVP. The interaction of poly-Arg with colloidal silver was confirmed with UV-Vis spectral shifts, where the absorbance peaks were shifted from 400 to 552 nm for yellow colloidal silver, while the blue silver peak shifted from 700 to 624 nm after the addition of poly-Arg (Figure 6).

**Figure 7:**
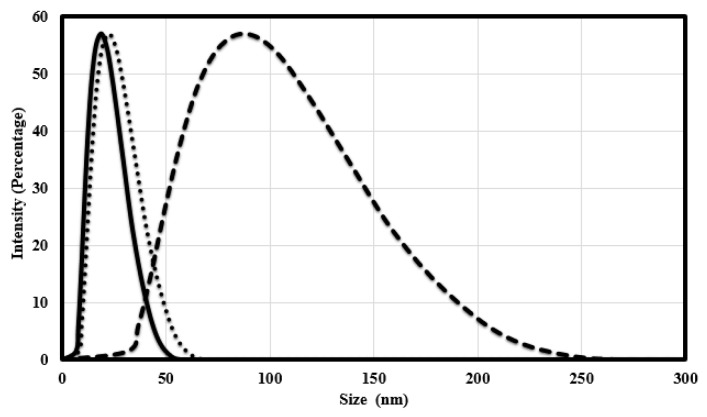
The particle size distribution profile of spherical silver nanoparticles coated with citrate (**─**), PVP (**∙∙∙**) and poly-Arg-PVP (**- -**) determined by dynamic light scattering.

**Figure 8:**
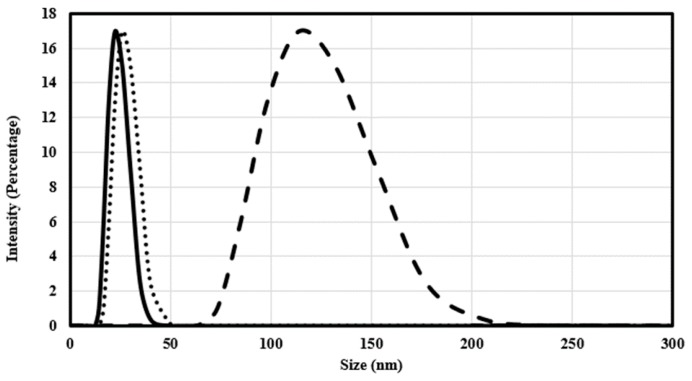
The particle size distribution profile of prismatic silver nanoparticles coated with citrate PVP (**∙∙∙**) and poly-Arg-PVP (**- -**) as determined by dynamic light scattering.

Using dynamic light scattering measurements, the mean diameter of spherical silver coated with PVP and poly-Arg-PVP was determined to be 23.56 nm and 90.75 nm, respectively (Figure 7). The mean diameters of prismatic silver coated with PVP and poly-Arg-PVP was 24.5 nm and 114 nm, respectively (Figure 8). Clearly, the PVP had a very minor effect on the hydrodynamic diameter, but the presence of poly-Arg increased the apparent size of the composite by a factor of approximately four. 

**Figure 9:**
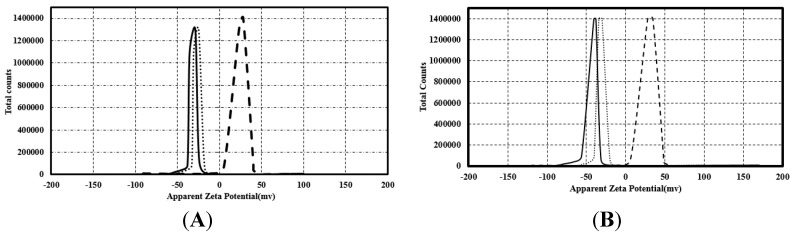
The apparent zeta potential (mV) of spherical (**A**) and prismatic (**B**) silver nanoparticles coated with citrate (**─**), PVP (**∙∙∙**) and poly-Arg-PVP (**- -**).

The electrostatic interaction of the nanoparticles can be controlled by the variation in their surface charge, which is determined by measuring the zeta potential of these particles. The zeta potential of spherical citrate and PVP capped AgNPs was −38 mV and −30 mV, respectively (Figure 9A), while that of prismatic citrate and PVP capped AgNPs was −29.28 and −25.7 mV, respectively. In contrast, the zeta potential of spherical AgNPs coated with poly-Arg-PVP was +34 mV (Figure 9B). The observed zeta potential value (−29.5 mV) for the citrate capped silver nanoparticles is close to the values reported in the literature for the stable suspensions comprised of nanoparticles with a negative charge on their surface [38]. The zeta potential of prismatic silver coated with poly-Arg-PVP was +28.7 mV, similar to the value for the spherical AgNPs with poly-Arg-PVP.

The enhancement of the stability of aqueous dispersions of the silver NPs is a major concern for practical use, since uncontrolled aggregation takes away the potential benefits of the nanomaterial. This stability enhancement can be obtained via two kinds of protecting mechanisms. The first one is based on steric repulsion, which displays a stabilizing effect with the assistance of polymers and non-ionic surfactants that are immediately adsorbed at the phase interphase [39]. The non-ionic surfactants are, in comparison to the polymers, adsorbed in a more compact mode at the surface of the NPs and convey an excellent stabilizing effect [40]. The stability of AgNPs coated with PVP was further confirmed using biological media such as phosphate buffered saline mixed with nutrient broth. This stabilization can be attributed to steric repulsion imparted by the adsorbed PVP playing a role in the prevention of aggregation [41].

### 2.3. Anti-Bacterial Effects

**Figure 10:**
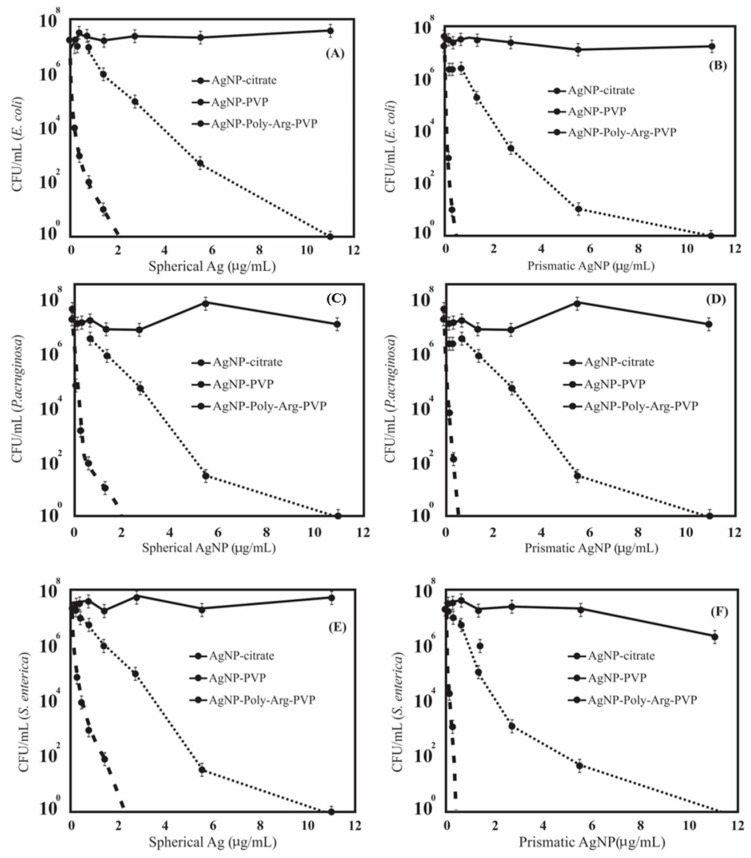
Shape dependent antimicrobial activity of silver nanoparticles coated with citrate, PVP and poly-Arg-PVP against *E. coli* (**A**,**B**), *P. aeruginosa* (**C**,**D**) and *S. enterica* (**E**,**F**).

Two different sets (spherical and prismatic) of three different types of colloidal silver nanoparticles (citrate capped, AgNP-PVP, AgNP-poly-Arg-PVP) were therefore produced. To compare the antibacterial effects of different AgNPs morphological shapes and coatings, the minimum bactericidal concentration (MBC) assay was carried out in liquid culture media using *E. coli*, *P. aeroginosa* and *S. enterica*, where the MBC is the lowest concentration of antimicrobial agent that completely inhibits growth. Concentrations of AgNPs used in this assay ranged from 11 to 0 µg/mL, and the initial bacterial inoculum was 2 × 10^7^ CFU/mL and the time and temperature of incubation was 24 h at 37 °C, respectively. The MBC measurement was performed in triplicate to confirm the value of MBC for each tested bacteria (Figure 10). The citrate capped AgNPs showed no inhibitory effect against all tested bacteria in the selected range of concentrations and both shapes of colloidal silvers. The likely explanation is the immediate aggregation of citrate capped AgNPs in the medium, which was confirmed using spectrophotometry by a loss of the peak absorbance. It has also been reported by Choi et al. [7] that the generation of aggregates leads to a loss of the antibacterial activity of nanoparticles in the medium of dispersion. The inhibitory effect of AgNPs was more prominent in our study, with PVP capped nanoparticles being compared to citrate ones, although the inhibitory action was found to be 50% more when colloidal silver was first coated with poly-Arg and subsequently stabilized with PVP.

The most plausible explanation for the enhanced activity of AgNP-poly-Arg-PVP could be the electrostatic interactions between negatively charged bacterial cells and the positively charged silver nanoparticles coated with poly-Arg. In previous studies with different materials, the superiority of the positively charged AgNPs over the negatively charged particles, in terms of the antibacterial activity, was demonstrated [20,34]. The zeta potential study of AgNP-poly-Arg-PVP nanomaterials showed positive values of +34 and +28.7 mV for spherical AgNPs and prismatic AgNPs, respectively (Figure 9). Zeta potential is an essential parameter for the indication of stability and charge for aqueous AgNPs suspensions. A minimum of ±30 mV zeta potential is required for the indication of a stable nano-suspension [37], which is very close to the values obtained in this work. Apart from obtaining favorable positive charges by coating with poly-Arg, it may also have functional properties that aid in the interaction with the cells [42].

Silver nanoprisms showed a greater inhibition activity against bacteria as compared to spherical nanoparticles. As the results show, the MBC value of the silver nanoprisms capped with poly-Arg-PVP was 0.65 μg/mL lower than the spherical ones, which is 2.7 μg/mL (Figure 10). These differences can be explained as demonstrated in other work [11,43] where the authors concluded that the nanocrystals with a basal plane had the strongest activity against the bacteria due to the high-atom-density facets. Thus, a high antibacterial activity of nanoprisms was found when compared to spherical NPs and their composites in this study. Therefore, from this study, it is suggested that the silver nanoprisms having very sharp vertexes and sharp edges were more effective in damaging the bacterial cell.

In another study, silver nanoparticles with mean size of 16 nm were completely cytotoxic for *E. coli* at a relatively high concentration of 60 mg/mL [44] but in this work poly-Arg-PVP capped prismatic AgNPs showed cytotoxic effects at a much lower concentration of 0.65 μg/mL against *E. coli*, *S. enterica* and *P. aeruginosa*. The results shown in Figure 10 and comparisons with literature indicate that the poly-Arg-PVP coating plays a significant role in enhancing the antimicrobial effect. Therefore, this was examined visually using TEM.

### 2.4. TEM Analysis of Silver NPs Interactions with Bacterial Wall

**Figure 11:**
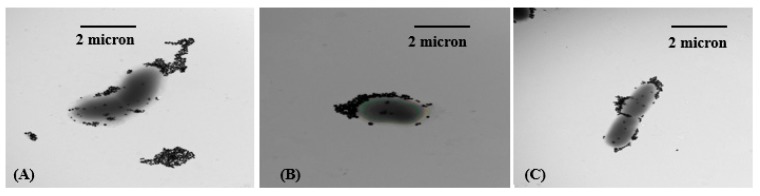
Representative TEM images of (**A**) *E. coli* (**B**) *P. aeruginosa* (**C**) *S. enterica* after treatment with spherical silver nanoparticles coated with poly-Arg-PVP.

**Figure 12:**
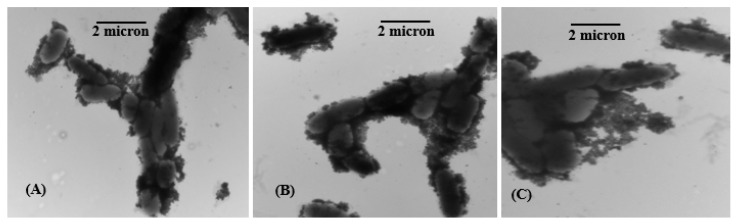
Representative TEM images of (**A**) *E. coli* (**B**) *P. aeruginosa* (**C**) *S. enterica* after treatment with prismatic silver nanoparticles coated with poly-Arg-PVP.

*E. coli*, *S. enterica* and *P. aeruginosa* treated with silver nanoparticles were prepared for TEM imaging in order to study the nature of the antibacterial interactions. As observed in TEM micrographs (Figure 11 and Figure 12), the poly-Arg-PVP-capped prismatic AgNPs were very strongly associated with the cell surfaces (Figure 12), especially as compared to the spherical AgNPs (Figure 11) where the number of attached nanoparticles was significantly smaller. This observation supports the hypothesis that the poly-Arg coating enhances the attraction to the cell surface through some combination of electrostatic and steric effects.

### 2.5. Mammalian Cell Cytotoxicity Evaluation

To provide some initial indications of the potential use of coated NPs in vivo, their impact on mammalian cell viability was measured using a panel of highly purified and well-characterized AgNPs with a specific focus on shape and capping ligand effects. The mechanism of toxicity was explored using the 3-(4,5-Dimethylthiazol-2-Yl)-2,5-Diphenyltetrazolium Bromide (MTT assay), based on an evaluation of the activity of mitochondrial dehydrogenases. Spherical and prismatic silver NPs coated with citrate, PVP, and poly-Arg-PVP were compared and AgNPs inhibited the viability of the HeLa cancer cell lines in a dose dependent manner. Cytotoxic activity was extremely sensitive to the shape and capping of the nanoparticles, and the viability measurements considerably decreased with increasing doses (0.02–11 μg/mL). 

**Figure 13:**
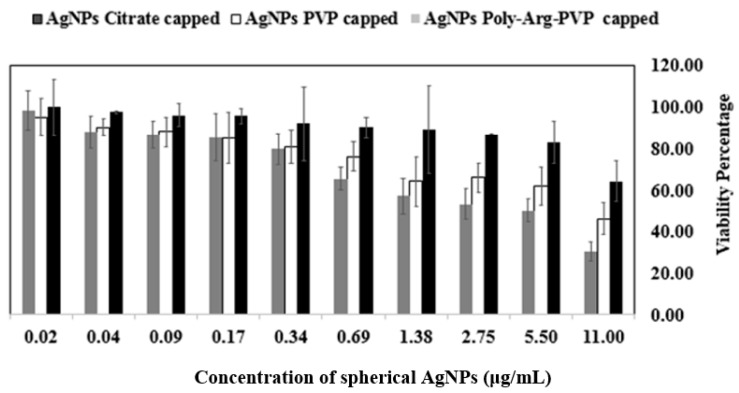
Cell viability of HeLa cell lines after treatment with spherical AgNPs coated with citrate, PVP and poly-Arg-PVP.

**Figure 14:**
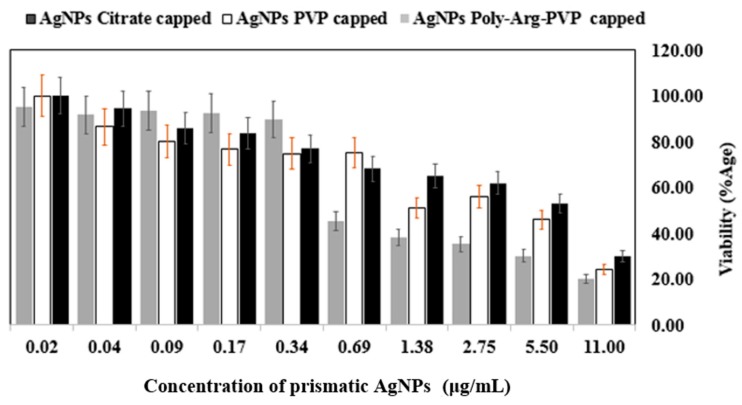
Cell viability of HeLa cell lines after treatment with prismatic AgNPs coated with citrate, PVP and poly-Arg-PVP.

Results showed the percentage viability of HeLa cells at various concentrations of AgNPs (from 0.02 to 11 μg/mL). Spherical AgNPs capped with citrate showed 64–100% viability, while PVP-capped viabilities ranged from 46–95% and poly-Arg-PVP capped from 30–98% at the same concentrations (Figure 13). For prismatic AgNPs, the citrate capped AgNPs showed 30–100%, while PVP-coated ranged from 24–100%, and poly-Arg-PVP coated 20–95% at the concentrations under study (Figure 14). Therefore, the results showed that prismatic AgNPs coated with poly-Arg-PVP have increased cytotoxic effects as compared to spherical AgNPs coated with poly-Arg-PVP. Statistical significance was determined with Student’s *t*-test using a one-way Analysis of Variance (ANOVA). Where ANOVA is the statistical method of analysis in which the variation in two set of groups were compared. Poly-Arg-PVP capped prismatic silver NPs showed a statistically significant reduction in cell viability (*p* < 0.05) when compared with poly-Arg-PVP capped spherical silver NPs from 11 to 0.69 µg/mL. It was reported that a decrease in the viability of bronchial BEAS-2B cell line was observed upon 24 h exposure to 20 nm citrate-coated, PVP coated AgNPs at 6.25–50 µg/mL [45]. The similar sized AgNPs in the present study, when capped with poly-Arg and protected with PVP, showed cell death at a much lower concentration range of 0.69 to 11 µg/mL. 

## 3. Discussion

Different shapes of AgNPs were prepared by a chemical reduction method. Size and shape were characterized by UV-vis spectroscopy, TEM and EDX. Spherical silver nanoparticles were synthesized with an average diameter of 20 nm, and silver nanoprisms of a similar size with sharp edges and vertexes were prepared in water at room temperature in the presence of hydrogen peroxide.

A novel composite was designed in this study based on a natural cationic polymer, namely poly-L-arginine. Prismatic AgNP-poly-Arg-PVP showed better antibacterial activity as compared to spherical AgNP-poly-Arg-PVP, with broad-spectrum antibacterial activity against *Escherichia coli*, *Pseudomonas aeruginosa*, and *Salmonella enterica*. The silver nanoparticles showed excellent antimicrobial activity and also had significant cytotoxic effects against an in vitro HeLa cancer cell line. Specific mechanisms of action remain to be explored in more detail.

## 4. Materials and Methods

Silver nitrate (AgNO_3_, 99.99%), trisodium citrate dihydrate (C_6_H_5_O_7_Na_3_·2H_2_O, 99.99%), sodium borohydride (NaBH_4_, 99.99%), hydrogen peroxide (H_2_O_2_ 30%), HNO_3_, polyvinylpyrrolidone (PVP; *MW* = 40,000), Poly-L-arginine (poly-Arg; *MW* = 5000–15,000), Cetrimide agar, and MacConkey agar were purchased from Sigma Aldrich (Oakville, ON, Canada). Agar (Eosin Methylene Blue Agar) was purchased from BD Biosciences (Mississauga, ON, Canada). *Escherichia coli* (ATCC PTA-4752), *Salmonella enterica* (ATCC BAA-1604) and *Pseudomonas aeruginosa* (ATCC 15442) were obtained from Cedarlane Laboratories, in Burlington, Ontario, Canada, and API (BioMerieux, Marcy-l’Étoile, France) kits and oxidase test strips were purchased from VWR Canada (Mississauga, ON, Canada) to confirm the bacteria identities. Nutrient broth powder was purchased from Thermo Fisher Scientific Inc. (Mississauga, ON, Canada). Plate Count Agar (DifcoTM) was purchased from Becton Dickinson and Company (Mississauga, ON, Canada). All of the solutions were prepared in ultrapure water with resistivity 18 MΩ cm^−1^. HeLa cell lines were purchased from the American Type Culture Collection (Rockville, MD, USA) and maintained at 37 °C in an atmosphere of 5% O_2_ in Dulbecco’s modified Eagle’s medium (Welgene, Gyeongsan, Korea) containing 10% fetal bovine serum (Gibco, Gaithersburg, MD, USA). Piranha solution (30:70 *v*/*v* solution of 30% hydrogen peroxide and concentrated sulfuric acid) was used for cleaning of glassware. 

### 4.1. Synthesis of Citrate Capped Spherical Silver Nanoparticles

100 µL of 100 mM silver nitrate, 1.5 mL of 100 mM trisodium citrate were mixed and diluted to 100 mL with water in a flask. The solution was stirred constantly, and 1 mL of 0.1 M sodium borohydride was rapidly added. After 2 min, the colorless solution turned yellow. The resulting suspension was stored in the dark at 4 °C. 

### 4.2. Synthesis of Citrate Capped Silver Nanoprisms

100 µL of silver nitrate (100 mM), 100 mM trisodium citrate (1.5 mL), and 280 μL of 30% hydrogen peroxide were mixed and diluted to 100 mL with water in a flask. The solution was vigorously stirred for 10 min, and 1 mL of 0.1 M sodium borohydride was rapidly added. After 2 min, the colorless solution turned yellow and then rapidly darkened until a stable blue color was developed after approximately 5 minutes. The resulting suspension was stored in the dark at 4 °C. This procedure allowed for the silver nanoprisms to be easily synthesized on a large scale with a degree of high stability. The experiment could be completed in less than 1 hour if the solutions had been prepared in advance [36].

AgNPs were left for 24 h then nano-composites were prepared in the following way. A 0.1% *w*/*v* poly-Arg aqueous solution was prepared. Then, 5 mL poly-Arg (0.1%) solution was added to 5 mL of citrate capped AgNPs and the shift in the absorbance was observed spectrophotometrically. PVP (0.033%) was added to prevent the aggregation of Ag nanocomposites. To remove poly-Arg and PVP excess, the mixture was centrifuged in 1.5 mL polypropylene Eppendorf tubes at 6708 g for 20 min, the supernatant was removed, and fresh Milli-Q water was added. This washing procedure was repeated three times. The resulting coated nanoparticle dispersions were stable against aggregation upon storage for 1 month at 4 °C when stored in the dark. This was determined by spectrometry, which showed no significant change in absorbance or spectrum during this time period. 

UV-visible spectra were recorded with a 1 cm path length cuvette using an HP 8452a diode array UV-Vis spectrophotometer (Agilent Technologies Inc., Santa Clara, CA, USA). Deionized water was used as the reference sample to take the blank spectrum for all measurements. UV-Vis extinction spectra were recorded in absorbance mode (range 200–800 nm) at desired dilutions of silver colloids. The maximum absorbance and the shifts in the surface plasmon resonance were recorded after the addition of the desired amount of poly-Arg along with PVP, and PVP alone, in yellow and blue silver nanoparticles.

TEM characterization was performed using a Philips CM10 equipment (Amsterdam, The Netherlands). The samples were prepared by drop-coating onto a carbon coated copper grid (200 mesh), the aqueous solution of nanoparticles, and nanocomposites (AgNP-PVP and AgNP-poly-Arg-PVP), and air-drying for approximately 0.5 h. The bacterial samples were prepared by first mixing 0.5 mL of bacterial solution (10^7^ CFU/mL) with 0.5 mL of colloidal silver solution. The mixtures were then incubated to allow for complete interaction. The colloidal silver/bacteria complexes were centrifuged at 5000 rpm for 5 min at 20 °C to remove unreacted colloidal silver. The samples were wash twice using deionized (DI) water and finally re-suspended in 100 µL of DI water. To prepare the TEM grid, 20 µL of the solution was dropped onto the grid and allowed to dry for half an hour and imaged using TEM.

Particle sizes (hydrodynamic diameters), polydispersity index, and zeta potential were measured using a Zetasizer Nano ZS90 (Malvern Instruments Ltd., Malvern, UK) operating with a He–Ne laser. The results were the means of triplicate runs, and in each run, 10 measurements were made. A refractive index (RI) of 1.5 was used, and the viscosity of the sample was assumed to be the viscosity of the dispersant. The sample was vortexed, and then transferred into either 2 mL cuvettes or a 1 mL clear zeta potential cuvette (DTS1060, Malvern Instruments Ltd., Malvern, UK). The electrophoretic mobility of the sample was measured and converted into the zeta potential by applying the Henry equation. The data were collected and analyzed with the Dispersion Technology software 5.1 (Malvern) producing curves for the particles size as intensity distribution or diagrams for the zeta potential as a distribution versus total counts.

To measure the amount of silver present in the nanoparticles and nanocomposites, ICP-OES (Prodigy, Teledyne Leeman, Hudson, NH, USA) was used. Before measuring via ICP-OES, the AgNPs and AgNPs nanocomposites were digested using a strong oxidizing acid solution comprised of 2–5% nitric acid and H_2_O_2_ 30% in a 1:1 ratio to convert the nanoparticles into the ionic form. The digested samples were compared against standard calibration curves after correcting for the dilution factor to find out the amount of silver.

Each species of bacteria were grown in nutrient broth overnight separately at 37 °C. After 12 h of incubation, these strains were centrifuged in a 1.5 mL Eppendorf tube at 5000× *g*. The supernatant was discarded and the pellet was collected and re-suspended in fresh nutrient broth. The optical density of this stock solution of bacteria was adjusted to between 0.07 and 0.08 at 600 nm for use in the study. McFarland standards (0.5) were used as a reference to adjust the turbidity of bacterial suspensions so that the number of bacteria will be within a desired range to standardize microbial testing. The bacterial cell concentration was also verified with the plate count method, and the initial bacterial density was found to be 2 × 10^7^ CFU/mL.

Two-fold dilutions were performed independently using with 5 mM phosphate buffer saline for each type of nanoparticles and their composites. Each 0.5 mL of the bacterial suspensions from the stock solution was inoculated into the corresponding tubes containing different concentrations (11 to 0 μg mL^−1^) but the same volume of silver nanoparticles (0.5 mL). The methodology also included a positive control (tubes containing inoculum and nutrient media, devoid of nanoparticles) and a negative control (tubes containing Ag nanoparticles and nutrient media, devoid of inoculum). The time and temperature of incubation were 24 h and 37 °C, respectively. The mass of silver nanoparticles and nanocomposites was determined by ICP-OES.

The solution was then placed onto a rotary shaker at 90 rpm and maintained at 37 °C for 12 h. After incubation, each sample was further subjected to serial dilutions for viable count and 0.1 mL of the bacterial solution was transferred to the surface of an agar plate in a sterile environment, and the solution was spread with a sterile plastic rod to evenly cover the surface. Then, all of the agar plates were placed in an incubator for colony growth at 37 °C for 24 h. The minimum bactericidal concentration (MBC) was determined according to the lowest silver nanoparticles and nanocomposites concentration that inhibited the visible growth of microbes after incubation overnight. The entire procedure was repeated three times independently.

HeLa cells were cultured in Dulbecco’s Modified Eagle’s Medium supplemented with 10% foetal bovine serum albumin at 37 °C under 5% CO_2_ incubator. HeLa cells were harvested in the logarithmic phase with a mixture of 0.05% trypsin and 0.53 mM, Ethylenediaminetetraacetic acid (EDTA). The medium was replaced three times per week, and the cells were passaged at subconfluency. An aliquot of 100 μL of the cells prepared at a density of 5 × 10^3^ cells/mL was plated in each well of 96 well plates, and 0.1 mL isopropanol with 0.04 M HCl was added to each well and mixed thoroughly by repeated pipetting with a multichannel pipettor. Acidified isopropyl alcohol (0.04 M HCl in isopropanol) were added to solubilize the MTT formazan [46]. The isopropanol dissolved the formazan to give a homogeneous blue solution suitable for absorbance measurement. After culturing for 24 h, the medium was refreshed with AgNPs prepared at specific concentrations (11.00, 5.50, 2.75, 1.38, 0.69, 0.34, 0.17, 0.09, 0.04, and 0.02 μg/mL). After incubation for a further 24 h, the cells were collected and analyzed for viability, and then 25 μL of MTT (3-(4,5-dimethylthiazol-2-yl)-2,5-diphenyltetrazolium bromide) stock solution (5 mg/mL in PBS) was added to each well to achieve a final concentration of 1 mg/mL, with the exception of the controlled “blank” wells, where 25 μL of PBS was added. After incubation for another 2 h, 100 μL of the buffer (20% SDS in 50% DMF, pH = 4.7, prepared at 37 °C) was added to the wells and incubated for another 4 h at 37 °C. The absorbance was measured at 570 nm using a SpectraMax M3 microplate reader (VWR, Mississauga, ON, Canada). Cell viability was normalized to that of HeLa cells cultured in the cell media. Three repetitions were conducted for each concentration, and the results were calculated for the cell viability.

## References

[1] Krzyzewska I., Kyziol-Komosinska J., Rosik-Dulewska C., Czupiol J., Antoszczyszyn-Szpicka P. (2016). Inorganic nanomaterials in the aquatic environment: Behavior, toxicity, and interaction with environmental elements. Arch. Environ. Prot..

[2] Ahumada M., McLaughlin S., Pacioni N.L., Alarcon E.I. (2016). Spherical silver nanoparticles in the detection of thermally denatured collagens. Anal. Bioanal. Chem..

[3] Alarcon E.I., Griffith M., Udekwu K.I. (2015). Silver Nanoparticle Applications.

[4] Da Costa P.M., Loureiro L., Matos A.J.F. (2013). Transfer of multidrug-resistant bacteria between intermingled ecological niches: The interface between humans, animals and the environment. Int. J. Environ. Res. Public Health.

[5] Alarcon E.I., Udekwu K., Skog M., Pacioni N.L., Stamplecoskie K.G., González-Béjar M., Polisetti N., Wickham A., Richter-Dahlfors A., Griffith M. (2012). The biocompatibility and antibacterial properties of collagen-stabilized, photochemically prepared silver nanoparticles. Biomaterials.

[6] Xiu Z.-M., Ma J., Alvarez P.J.J. (2011). Differential effect of common ligands and molecular oxygen on antimicrobial activity of silver nanoparticles versus silver ions. Environ. Sci. Technol..

[7] Choi J.Y., Yoo J.Y., Kwak H.S., Nam B.U., Lee J. (2005). Role of polymeric stabilizers for drug nanocrystal dispersions. Curr. Appl. Phys..

[8] Franci G., Falanga A., Galdiero S., Palomba L., Rai M., Morelli G., Galdiero M. (2015). Silver nanoparticles as potential antibacterial agents. Molecules.

[9] Helmlinger J., Sengstock C., Gross-Heitfeld C., Mayer C., Schildhauer T.A., Koeller M., Epple M. (2016). Silver nanoparticles with different size and shape: Equal cytotoxicity, but different antibacterial effects. RSC Adv..

[10] Lu W., Yao K., Wang J., Yuan J. (2015). Ionic liquids-water interfacial preparation of triangular Ag nanoplates and their shape-dependent antibacterial activity. J. Colloid Interface Sci..

[11] Pal S., Tak Y.K., Song J.M. (2007). Does the antibacterial activity of silver nanoparticles depend on the shape of the nanoparticle? A study of the gram-negative bacterium. Escherichia coli. Appl. Environ. Microbiol..

[12] Peretyazhko T.S., Zhang Q., Colvin V.L. (2014). Size-controlled dissolution of silver nanoparticles at neutral and acidic pH conditions: Kinetics and size changes. Environ. Sci. Technol..

[13] Shaban S.M., Aiad I., El-Sukkary M.M., Soliman E.A., El-Awady M.Y. (2015). Preparation of capped silver nanoparticles using sunlight and cationic surfactants and their biological activity. Chin. Chem. Lett..

[14] Zhu Q.-L., Xu Q. (2014). Metal-organic framework composites. Chem. Soc. Rev..

[15] Alarcon E., Vulesevic B., Argawal A., Ross A., Bejjani P., Podrebarac J., Ravichandran R., Phopase J., Suuronen E., Griffith M. (2016). Coloured cornea replacements with anti-infective properties: Expanding the safe use of silver nanoparticles in regenerative medicine. Nanoscale.

[16] Alarcon E.I., Udekwu K.I., Noel C.W., Gagnon L.B.-P., Taylor P.K., Vulesevic B., Simpson M.J., Gkotzis S., Islam M.M., Lee C.-J. (2015). Safety and efficacy of composite collagen–silver nanoparticle hydrogels as tissue engineering scaffolds. Nanoscale.

[17] Allison S., Ahumada M., Andronic C., McNeill B., Variola F., Griffith M., Ruel M., Hamel V., Liang W., Suuronen E.J. (2017). Electroconductive nanoengineered biomimetic hybrid fibers for cardiac tissue engineering. J. Mater. Chem. B.

[18] McLaughlin S., Ahumada M., Franco W., Mah T.-F., Seymour R., Suuronen E.J., Alarcon E.I. (2016). Sprayable peptide-modified silver nanoparticles as a barrier against bacterial colonization. Nanoscale.

[19] Poblete H., Agarwal A., Thomas S.S., Bohne C., Ravichandran R., Phospase J., Comer J., Alarcon E.I. (2015). New insights into peptide–silver nanoparticle interaction: Deciphering the role of cysteine and lysine in the peptide sequence. Langmuir.

[20] Abbaszadegan A., Ghahramani Y., Gholami A., Hemmateenejad B., Dorostkar S., Nabavizadeh M., Sharghi H. (2015). The effect of charge at the surface of silver nanoparticles on antimicrobial activity against gram-positive and gram-negative bacteria: A preliminary study. J. Nanomater..

[21] Radomski A., Jurasz P., Alonso-Escolano D., Drews M., Morandi M., Malinski T., Radomski M.W. (2005). Nanoparticle-induced platelet aggregation and vascular thrombosis. Br. J. Pharmacol..

[22] Yue Z.-G., Wei W., Lv P.-P., Yue H., Wang L.-Y., Su Z.-G., Ma G.-H. (2011). Surface charge affects cellular uptake and intracellular trafficking of chitosan-based nanoparticles. Biomacromolecules.

[23] Silhavy T.J., Kahne D., Walker S. (2010). The bacterial cell envelope. Cold Spring Harb. Perspect. Biol..

[24] Munch D., Sahl H.G. (2015). Structural variations of the cell wall precursor lipid II in Gram-positive bacteria—Impact on binding and efficacy of antimicrobial peptides. Biochim. Biophys. Acta.

[25] Salvioni L., Galbiati E., Collico V., Alessio G., Avvakumova S., Corsi F., Tortora P., Prosperi D., Colombo M. (2017). Negatively charged silver nanoparticles with potent antibacterial activity and reduced toxicity for pharmaceutical preparations. Int. J. Nanomed..

[26] Stephens E., Tauran Y., Coleman A., Fitzgerald M. (2015). Structural requirements for anti-oxidant activity of calix [n] arenes and their associated anti-bacterial activity. Chem. Commun..

[27] Jung W.K., Koo H.C., Kim K.W., Shin S., Kim S.H., Park Y.H. (2008). Antibacterial activity and mechanism of action of the silver ion in *Staphylococcus aureus* and *Escherichia coli*. Appl. Environ. Microbiol..

[28] Nel A.E., Madler L., Velegol D., Xia T., Hoek E.M.V., Somasundaran P., Klaessig F., Castranova V., Thompson M. (2009). Understanding biophysicochemical interactions at the nano-bio interface. Nat. Mater..

[29] Thill A., Zeyons O., Spalla O., Chauvat F., Rose J., Auffan M., Flank A.M. (2006). Cytotoxicity of CeO_2_ Nanoparticles for *Escherichia coli*. Physico-Chemical Insight of the Cytotoxicity Mechanism. Environ. Sci. Technol..

[30] Herce H.D., Garcia A.E., Litt J., Kane R.S., Martin P., Enrique N., Rebolledo A., Milesi V. (2009). Arginine-rich peptides destabilize the plasma membrane, consistent with a pore formation translocation mechanism of cell-penetrating peptides. Biophys. J..

[31] Joliot A., Prochiantz A. (2004). Transduction peptides: From technology to physiology. Nat. Cell Biol..

[32] Tang H., Yin L., Kim K.H., Cheng J. (2013). Helical poly (arginine) mimics with superior cell-penetrating and molecular transporting properties. Chem. Sci..

[33] Li J.G., Liu S.P., Lakshminarayanan R., Bai Y., Pervushin K., Verma C., Beuerman R.W. (2013). Molecular simulations suggest how a branched antimicrobial peptide perturbs a bacterial membrane and enhances permeability. Biochim. Biophys. Acta.

[34] Silva T., Pokhrel L.R., Dubey B., Tolaymat T.M., Maier K.J., Liu X. (2014). Particle size, surface charge and concentration dependent ecotoxicity of three organo-coated silver nanoparticles: Comparison between general linear model-predicted and observed toxicity. Sci. Total Environ..

[35] Panzarasa G. (2015). Just what is it that makes silver nanoprisms so different, so appealing?. J. Chem. Educ..

[36] Haes A.J., Van Duyne R.P. (2002). A nanoscale optical biosensor: Sensitivity and selectivity of an approach based on the localized surface plasmon resonance spectroscopy of triangular silver nanoparticles. J. Am. Chem. Soc..

[37] Shameli K., Bin Ahmad M., Jazayeri S.D., Sedaghat S., Shabanzadeh P., Jahangirian H., Mahdavi M., Abdollahi Y. (2012). Synthesis and characterization of polyethylene glycol mediated silver nanoparticles by the green method. Int. J. Mol. Sci..

[38] Kittler S., Greulich C., Diendorf J., Koeller M., Epple M. (2010). Toxicity of silver nanoparticles increases during storage because of slow dissolution under release of silver ions. Chem. Mater..

[39] Kvitek L., Panacek A., Soukupova J., Kolar M., Vecerova R., Prucek R., Holecova M., Zboril R. (2008). Effect of surfactants and polymers on stability and antibacterial activity of silver nanoparticles (NPs). J. Phys. Chem. C.

[40] Marambio-Jones C., Hoek E.M.V. (2010). A review of the antibacterial effects of silver nanomaterials and potential implications for human health and the environment. J. Nanopart. Res..

[41] Huynh K.A., Chen K.L. (2011). Aggregation kinetics of citrate and polyvinylpyrrolidone coated silver nanoparticles in monovalent and divalent electrolyte solutions. Environ. Sci. Technol..

[42] Shlar I., Poverenov E., Vinokur Y., Horev B., Droby S., Rodov V. (2015). High-throughput screening of nanoparticle-stabilizing ligands: Application to preparing antimicrobial curcumin nanoparticles by antisolvent precipitation. Nano-Micro Lett..

[43] Morones J.R., Elechiguerra J.L., Camacho A., Holt K., Kouri J.B., Ramirez J.T., Yacaman M.J. (2005). The bactericidal effect of silver nanoparticles. Nanotechnology.

[44] Raffi M., Hussain F., Bhatti T.M., Akhter J.I., Hameed A., Hasan M.M. (2008). Antibacterial characterization of silver nanoparticles against *E. coli* ATCC-15224. J. Mater. Sci. Technol..

[45] Wang X., Ji Z.X., Chang C.H., Zhang H.Y., Wang M.Y., Liao Y.P., Lin S.J., Meng H., Li R.B., Sun B.B. (2014). Use of coated silver nanoparticles to understand the relationship of particle dissolution and bioavailability to cell and lung toxicological potential. Small.

[46] Green L.M., Reade J.L., Ware C.F. (1984). Rapid colormetric assay for cell viability—Application to the quantitation of cyto-toxic and growth inhibitory lymphokines. J. Immunol. Methods.

